# Reliability of Measurements Performed by Community-Drawn Anthropometrists from Rural Ethiopia

**DOI:** 10.1371/journal.pone.0030345

**Published:** 2012-01-24

**Authors:** Berhan Ayele, Abaineh Aemere, Teshome Gebre, Zerihun Tadesse, Nicole E. Stoller, Craig W. See, Sun N. Yu, Bruce D. Gaynor, Charles E. McCulloch, Travis C. Porco, Paul M. Emerson, Thomas M. Lietman, Jeremy D. Keenan

**Affiliations:** 1 The Carter Center, Addis Ababa, Ethiopia; 2 Goncha Siso Enese Woreda Health Office, Gindewoin, Ethiopia; 3 Francis I. Proctor Foundation, University of California San Francisco, San Francisco, California, United States of America; 4 Department of Ophthalmology, University of California San Francisco, San Francisco, California, United States of America; 5 Department of Epidemiology and Biostatistics, University of California San Francisco, San Francisco, California, United States of America; 6 The Carter Center, Atlanta, Georgia, United States of America; 7 Institute for Global Health, University of California San Francisco, San Francisco, California, United States of America; State University of New York College at Oneonta, United States of America

## Abstract

**Background:**

Undernutrition is an important risk factor for childhood mortality, and remains a major problem facing many developing countries. Millennium Development Goal 1 calls for a reduction in underweight children, implemented through a variety of interventions. To adequately judge the impact of these interventions, it is important to know the reproducibility of the main indicators for undernutrition. In this study, we trained individuals from rural communities in Ethiopia in anthropometry techniques and measured intra- and inter-observer reliability.

**Methods and Findings:**

We trained 6 individuals without prior anthropometry experience to perform weight, height, and middle upper arm circumference (MUAC) measurements. Two anthropometry teams were dispatched to 18 communities in rural Ethiopia and measurements performed on all consenting pre-school children. Anthropometry teams performed a second independent measurement on a convenience sample of children in order to assess intra-anthropometrist reliability. Both teams measured the same children in 2 villages to assess inter-anthropometrist reliability. We calculated several metrics of measurement reproducibility, including the technical error of measurement (TEM) and relative TEM. In total, anthropometry teams performed measurements on 606 pre-school children, 84 of which had repeat measurements performed by the same team, and 89 of which had measurements performed by both teams. Intra-anthropometrist TEM (and relative TEM) were 0.35 cm (0.35%) for height, 0.05 kg (0.39%) for weight, and 0.18 cm (1.27%) for MUAC. Corresponding values for inter-anthropometrist reliability were 0.67 cm (0.75%) for height, 0.09 kg (0.79%) for weight, and 0.22 kg (1.53%) for MUAC. Inter-anthropometrist measurement error was greater for smaller children than for larger children.

**Conclusion:**

Measurements of height and weight were more reproducible than measurements of MUAC and measurements of larger children were more reliable than those for smaller children. Community-drawn anthropometrists can provide reliable measurements that could be used to assess the impact of interventions for childhood undernutrition.

## Introduction

Undernutrition remains an important problem for many developing countries. Wasting (low weight for height), stunting (low height for age), and underweight (low weight for age) contribute to many childhood illnesses and are risk factors for mortality [1]. The Millennium Development Goals have recognized the importance of undernutrition for development and have called for reductions in the prevalence of underweight children (Goal 1) and childhood mortality (Goal 4) [2]. Indices of undernutrition, such as weight, height, and middle upper arm circumference (MUAC) are therefore important outcome measures for government agencies and non-governmental organizations promoting nutrition and child health interventions [3].

Anthropometric assessment is especially important in poor rural areas of developing countries, where undernutrition is more severe [4], [5]. However, in rural areas, there is often a shortage of skilled personnel available for anthropometric monitoring, as community health workers are often occupied with other duties. Because the most important anthropometric measurements are relatively easy to master, community members without health care experience could potentially learn these skills and perform measurements for community-based monitoring. In this study, we trained community members in rural Ethiopia how to measure weight, height, and MUAC, and assessed the reproducibility of their measurements.

## Methods

### Ethics Statement

The study was registered with clinicaltrials.gov, numbers NCT00322972 and NCT01202331. The study had approval from the Committee for Human Research of the University of California, San Francisco, Emory University, and the Ethiopian Ministry of Science and Technology. The study was carried out in accordance with the Declaration of Helsinki and overseen by a Data Safety and Monitoring Committee appointed by the National Institutes of Health-National Eye Institute. Verbal informed consent in the local language was obtained from the guardian of all children. Verbal consent was approved by all institutional review boards, and was used due to the high prevalence of illiteracy in the study area.

### Study Design

This study describes the reproducibility of several secondary outcome measures (height, weight, and MUAC) from a series of cluster-randomized clinical trials performed in Goncha Siso Enese *woreda*, Amhara Region, Ethiopia. In the clinical trials, 72 *subkebeles* (government-defined subdistricts) were randomized to 1 of 6 different trachoma treatment strategies [6], [7], [8]. In March 2011 (58 months after the baseline visit) we offered anthropometric measurements to all children aged 0–5 years from 18 of these subkebeles.

We performed height, weight, and MUAC measurements using techniques recommended by the World Health Organization [9], [10]. Children were measured barefoot and with only light clothing. For all 3 anthropometric outcomes, the official measurement consisted of the median value of 3 independent replicate measurements. Children and/or equipment were adjusted between each of the replicate measurements.

### Height Measurements

To measure height, we used a portable measuring board (Shorr Productions, LLC, Olney, MD, USA), which was placed on a flat surface with the backboard supported by a tree or wall. Children were measured with the head, back, buttocks, and heels touching the backboard; heels together; knees extended; and head in the Frankfort horizontal plane. If a child could not cooperate sufficiently for a standing height measurement, the measuring board was placed on the ground, and the length measured with the same positioning. Measurements were taken to the nearest 0.1 cm.

### Weight Measurements

To measure weight, we used a Seca 874 scale (Seca GmbH & Co. KG, Hamburg, Germany), taking care to position the scale with all 4 feet of the scale touching the ground. No platform was used underneath the scale. We taped 2 footprints on the scale and asked children to stand on the footprints, ensuring that their weight was evenly distributed. For younger children, we used the taring function of the scale, in which the child's guardian stepped on the scale without the child, the scale was zeroed, and then the child was handed to the guardian. Weight measurements were recorded to the nearest 0.01 kg. Two 4.5 kg test weights were measured after every 10^th^ child to assess drift in the weight measurements over time. We performed 2 measurements: one with only the first test weight, and another with both test weights.

### MUAC Measurements

To measure MUAC, we used non-stretch MUAC tapes produced for clinical studies in Bangladesh (generously provided by A. Labrique) [11]. The child's right arm was flexed to 90° at the elbow, and the midpoint between the lateral acromion and distal olecranon was identified and marked. The arm was then relaxed, the MUAC strip was placed snugly around the marked midpoint of the arm, and the measurement was recorded to the nearest 0.1 cm.

### Anthropometry Training

The local health office referred 22 individuals for training. These individuals were largely farmers by profession, and had little or no knowledge of anthropometry. We trained potential anthropometrists over a 2-day period before the assessments began, using materials from the World Health Organization (WHO) [12]. During the first day of training, we showed a video produced by the WHO that described each of the anthropometric measurements [13]. The investigators demonstrated each anthropometric technique in front of the entire group, and reviewed potential sources for error. We then established several stations with the anthropometry equipment, and trainees practiced taking weight and MUAC measurements on each other, and height/length measurements on household objects. The investigators monitored each group, correcting trainees in their technique when necessary. On the second day of training, we asked potential anthropometrists to perform a series of test measurements on known heights, weights, and circumferences; the 6 individuals who performed these measurements most accurately were invited to be anthropometry team members. Besides the formal training session, we also provided daily supervision and feedback for both anthropometry teams while in the field.

Anthropometry teams were comprised of 3 individuals: a registrar, a measurer, and a recorder. In addition, an observer from the University of California, San Francisco or The Carter Center, Ethiopia was assigned to each team. The registrar was responsible for recruiting all under-5 year-old children and assigned a 6-digit random number sticker to each child who presented for anthropometry. The measurer led the child through a series of 3 anthropometric tests: height, then weight, then MUAC. Measurers performed each measurement in triplicate, calling out each measurement to the recorder. The recorder, in addition to transcribing measurements, also assisted in positioning children for each test. The teams were comprised of the same 3 individuals for the entire study visit. Team members were free to perform any of the team functions, and could switch positions as they wished. The role of the observer was to watch the measurer, and independently record a measurement before the measurer had called out any measurement.

### Repeat Measurements

We performed 3 types of repeated measurements in order to assess reliability. First, the measurements for all children were recorded by both the measurer-recorder team and by an independent observer. The observer wrote the measurement silently before the measurer called out his reading to the recorder, thus maintaining masking of both sets of measurements. Second, intra-anthropometrist agreement was assessed by sending a convenience sample of children for repeat registration and a new random number sticker immediately after completion of one round of anthropometric tests. These children were then re-measured by the team. We required that at least 4 other children be measured between the first and second measurements, to prevent the anthropometrists from recalling their previous measurement. Third, to measure inter-anthropometrist agreement, all children from 2 of the subkebeles were measured by both anthropometry teams on the same day. The teams set up approximately 50 meters away from one another, preventing each team from hearing the other's measurements. Repeat measurements were conducted identically to the first measurement (i.e., in triplicate, with the median used as the official value).

### Statistical methods

We performed several tests of reliability. Technical error of measurement (TEM) is the square root of the measurement error variance, which is the same as the within-subject standard deviation when repeated measurements are taken [14]. TEM is expressed in the units of the measurement, making comparisons of different tests difficult. Therefore, we also calculated the relative TEM, which is the TEM divided by the mean of all measurements [15]. We calculated the coefficient of reliability, which is numerically the same as the intraclass correlation coefficient (the between-subject variance divided by the total variance). The coefficient of reliability reflects the proportion of total between-subject variance not due to measurement error [14]. Finally, we calculated the repeatability, which is the TEM multiplied by 2.77 [16]. The repeatability coefficient reflects how different any 2 replicate measurements could be by chance alone; for 95% of subjects, the difference between 2 measurements will be less than or equal to the repeatability coefficient. Note that these metrics are all related, and are simply different ways to express the variability between repeated measurements.

We calculated estimates of intra-anthropometrist reliability for the children who had repeat measurements by the same anthropometrist, inter-anthropometrist reliability for the children who had repeat measurements by different anthropometry teams, and inter-observer reliability for all children. We calculated all statistics using the median of the 3 triplicate measurements as a single estimate of the measurement. We report intra-anthropometrist reliability separately for each measurer. In order to estimate the overall intra-anthropometrist reliability, we also performed analyses with aggregated data.

Bland-Altman plots were constructed to assess intra-anthropometrist and inter-anthropometrist reproducibility by plotting the mean of the 2 median measurements versus the percentage difference between the 2 median measurements (calculated as the difference divided by the mean). On each graph, we also plotted the mean percentage difference (also called the bias, since this is the tendency for one measurement to exceed the other), and the 95% limits of agreement (calculated as the mean percentage difference ±1.96 multiplied by the standard deviation of the percentage differences) [16]. The limits of agreement provide an estimate of reproducibility: the percentage difference between the 2 replicate measurements will lie between these limits for 95% of the measurement pairs. We dealt with heteroskedasticity in the Bland-Altman plots by stratifying the pairs of measurements into quartiles (based on the mean of the 2 measurements), and calculating the TEM and %TEM separately for each quartile.

We determined whether taking the median of 3 measurements reduced measurement error by calculating the %TEM for the first of the 3 measurements, the median of the 3 measurements, and the mean of the 3 measurements. We tested whether the scales experienced any measurement drift throughout the study by plotting the median measurement of each of the standard test weights over time. We assessed whether these test weight measurements changed over time in a linear regression adjusted for the scale, test weight set, and anthropometry team. Autocorrelation was assessed with the Wooldridge test for serial correlation [17]. We assessed the height and MUAC measurements for terminal digit preference by plotting the proportion of measurements with each of the 10 possible terminal digits, using values from only the first of the 3 replicate measures. To determine whether the proportion of measurements using each terminal digit was similar, we used the χ^2^ goodness of fit test from a multinomial regression with the terminal digit (0 through 9) as the outcome, accounting for community clustering. All statistical analyses were performed with Stata 10 (Statacorp, College Station, TX).

## Results

The 2 anthropometry teams monitored 606 children over 10 days. In 1 of the teams, the same person was the measurer for the entire study period (*N* = 328), whereas in the other team, all 3 team members functioned as the measurer at some point in the study (*N* = 152, 98, and 28, respectively). Of these 606 children, 594 had repeat measurements for height, weight, and MUAC documented by both the measurer-recorder team and the independent observer. 84 had repeat measurements performed by the same anthropometrist, and 89 separate children had repeat measurements performed by different anthropometry teams.

Each time the measurer-recorder team positioned and measured a child, an independent observer also recorded measurements. The agreement between these 2 records, which we call inter-observer reliability, is shown in Table 1 for the 594 children with complete data. In general, measurements between the anthropometry team and independent observer demonstrated excellent agreement. Note that in this study, inter-observer reliability does not capture any of the measurement variability associated with positioning the child.

**Table 1:** Inter-observer reliability for simultaneous measurements of 594 children in rural Ethiopia.

	Estimate (95% Confidence Interval)		
Metric	Height	Weight	MUAC
Mean	88.6 cm (87.7 to 89.5)	11.89 kg (11.66 to 12.11)	14.1 cm (14.0 to 14.2)
TEM	0.10 cm (0.09 to 0.10)	0.01 kg (0.01 to 0.01)	0.08 cm (0.07 to 0.08)
%TEM	0.11% (0.11 to 0.12)	0.07% (0.07 to 0.07%)	0.56% (0.53 to 0.59)
Reliability (ICC)	>0.999 (>0.999 to >0.999)	>0.999 (>0.999 to >0.999)	0.995 (0.994 to 0.995)
Repeatability	0.27 cm (0.26 to 0.29)	0.02 kg (0.02 to 0.02)	0.22 cm (0.21 to 0.23)

TEM = technical error of measurement; %TEM = relative TEM; ICC = intraclass correlation coefficient; MUAC = middle upper arm circumference.

**Figure 1:**
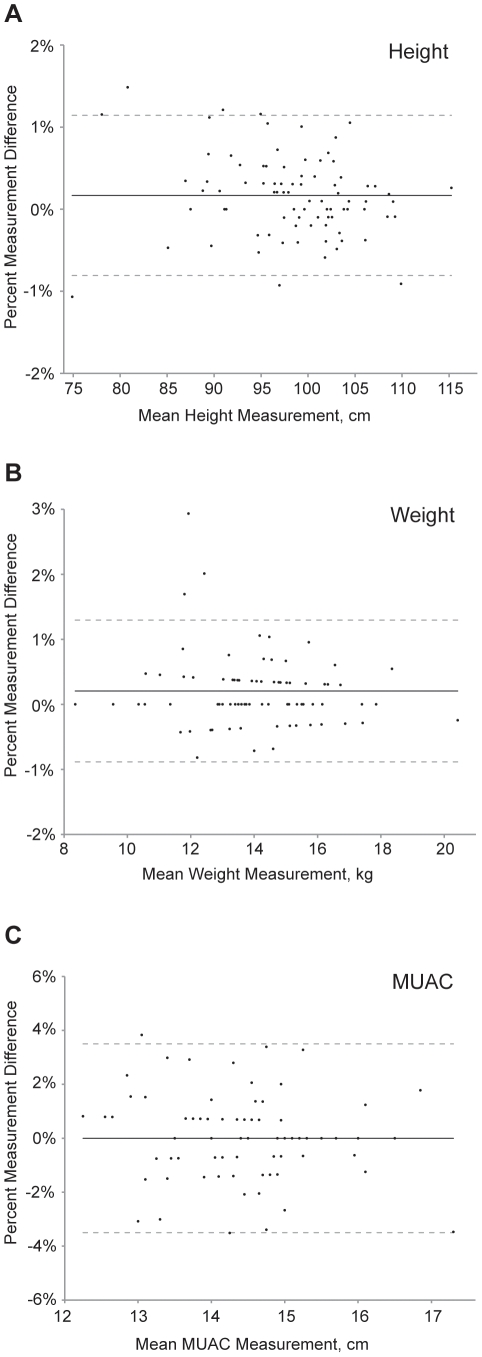
Bland-Altman plots depicting intra-anthropometrist agreement. Plots shown for measurements of (A) height, (B) weight, and (C) middle upper arm circumference in 84 children aged 0–5 years in a community-based study in Ethiopia. The solid horizontal line represents the mean percent difference between the measurements, and the dashed lines represent the 95% limits of agreement.

Estimates of intra-anthropometrist reliability for height, weight, and MUAC are shown in Table 2, separately for each measurer. All height measurements in intra-anthropometrist reliability calculations reflect standing height (as opposed to length). Intra-reliability metrics were generally similar for the individual graders. To estimate the overall intra-anthropometrist reliability, we also performed calculations using aggregated data (Table 2). The degree of intra-anthropometrist measurement error did not appear to depend on the magnitude of the measurement, as depicted in Bland-Altman plots (Figure 1).

**Table 2:** Intra-anthropometrist reliability for repeated measurements of 84 children in rural Ethiopia.

		Estimate (95% Confidence Interval)		
Metric	Observer	Height	Weight	MUAC
Mean	1	98.1 cm (95.6 to 100.6)	13.93 kg (13.22 to 14.65)	14.3 cm (14.0 to 14.6)
	2	98.3 cm (96.1 to 100.6)	14.07 kg (12.44 to 14.69)	14.5 cm (14.1 to 14.8)
	3	99.2 cm (93.7 to 104.8)	14.42 kg (12.83 to 16.01)	14.0 cm (13.0 to 15.0)
	All	98.3 cm (96.8 to 99.9)	14.04 kg (13.60 to 14.47)	14.4 cm (14.1 to 14.6)
TEM	1	0.38 cm (0.30 to 0.47)	0.07 kg (0.05 to 0.08)	0.21 cm (0.16 to 0.26)
	2	0.32 cm (0.25 to 0.39)	0.04 kg (0.03 to 0.05)	0.16 cm (0.13 to 0.20)
	3	0.29 cm (0.14 to 0.44)	0.02 kg (0.01 to 0.03)	0.12 cm (0.06 to 0.18)
	All	0.35 cm (0.29 to 0.40)	0.05 kg (0.05 to 0.06)	0.18 cm (0.15 to 0.21)
%TEM	1	0.39% (0.30 to 0.48)	0.49% (0.38 to 0.61)	1.48% (1.14 to 1.83)
	2	0.33% (0.26 to 0.40)	0.31% (0.24 to 0.38)	1.12% (0.87 to 1.36)
	3	0.29% (0.14 to 0.45)	0.13% (0.06 to 0.20)	0.85% (0.40 to 1.30)
	All	0.35% (0.30 to 0.41)	0.39% (0.33 to 0.45)	1.27% (1.08 to 1.46)
Reliability	1	0.997 (0.996 to 0.999)	0.999 (0.998 to >0.999)	0.939 (0.900 to 0.978)
	2	0.998 (0.997 to 0.999)	>0.999 (0.999 to >0.999)	0.981 (0.969 to 0.992)
	3	0.998 (0.994 to >0.999)	>0.999 (>0.999 to >0.999)	0.989 (0.971 to >0.999)
	All	0.998 (0.997 to 0.999)	0.999 (0.999 to >0.999)	0.969 (0.956 to 0.982)
Repeatability	1	1.06 cm (0.82 to 1.31)	0.19 kg (0.15 to 0.23)	0.59 cm (0.45 to 0.72)
	2	0.89 cm (0.70 to 1.08)	0.12 kg (0.09 to 0.15)	0.45 cm (0.35 to 0.54)
	3	0.81 cm (0.38 to 1.23)	0.05 kg (0.02 to 0.08)	0.33 cm (0.16 to 0.50)
	All	0.96 cm (0.82 to 1.11)	0.15 kg (0.13 to 0.17)	0.50 cm (0.43 to 0.58)

Reliability calculations are shown separately for each of the 3 measurers in the study, and also using aggregated data from all 3 measurers.

TEM = technical error of measurement; %TEM = relative TEM; ICC = intraclass correlation coefficient; MUAC = middle upper arm circumference.

**Figure 2:**
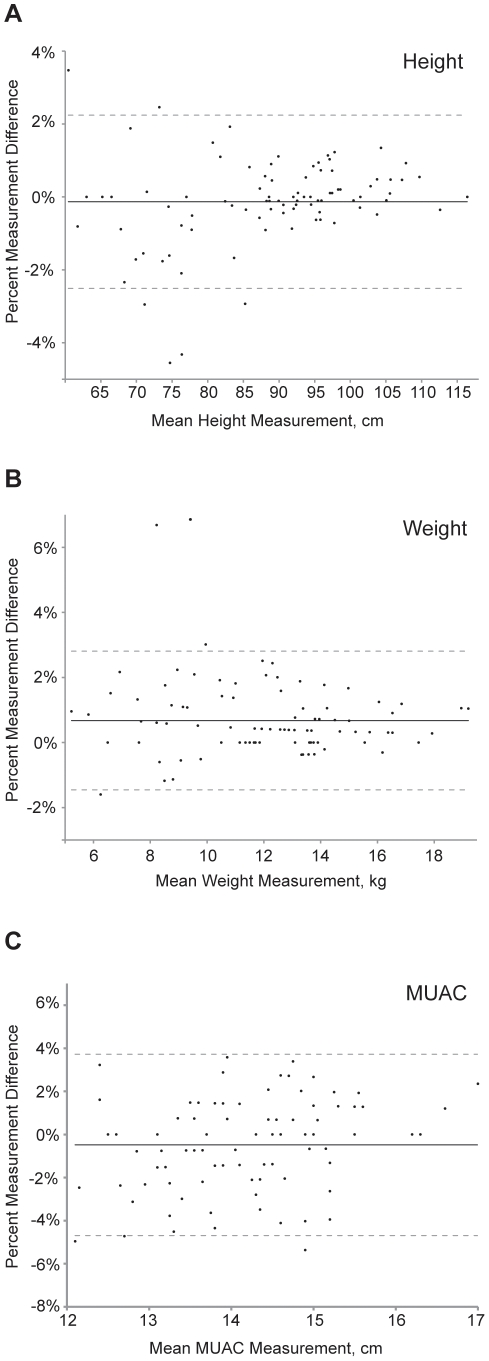
Bland-Altman plots depicting inter-anthropometrist agreement. Plots shown for measurements of (A) height, (B) weight, and (C) middle upper arm circumference in 89 children aged 0–5 years in a community-based study in Ethiopia. The solid horizontal line represents the mean percent difference between the measurements, and the dashed lines represent the 95% limits of agreement.

Table 3 lists estimates of inter-anthropometrist reliability for 89 children with repeat measurements. Inter-anthropometrist measurement error was greater than the corresponding values for intra-anthropometrist reliability (compare with Table 2). Bland-Altman plots of inter-anthropometrist reliability are depicted in Figure 2; these plots suggested greater measurement error in larger compared to smaller children. To further investigate this, we stratified children into 4 quartiles for each of the anthropometric measures (Table 4), and we compared measurements from the 61 children who had standing height measured versus the 28 who had length measured (Table 5). We found increased measurement error in smaller children compared with larger children, and for length measurements compared with height measurements. Even in the strata with the largest measurement errors, the relative TEM was still less than 2% for each anthropometry metric.

**Table 3:** Inter-anthropometrist reliability for repeated measurements of 89 children in rural Ethiopia.

	Estimate (95% Confidence Interval)		
Metric	Height	Weight	MUAC
Mean	88.7 cm (86.0 to 91.4)	12.05 kg (11.40 to 12.69)	14.2 cm (14.0 to 14.4)
TEM	0.67 cm (0.57 to 0.76)	0.09 kg (0.08 to 0.11)	0.22 cm (0.18 to 0.25)
%TEM	0.75% (0.64 to 0.86)	0.79% (0.67 to 0.91)	1.53% (1.30 to 1.76)
Reliability (ICC)	0.997 (0.996 to 0.998)	0.999 (0.999 to 0.999)	0.954 (0.935 to 0.973)
Repeatability	1.85 cm (1.57 to 2.12)	0.26 kg (0.22 to 0.30)	0.60 cm (0.51 to 0.69)

TEM = technical error of measurement; %TEM = relative TEM; ICC = intraclass correlation coefficient; MUAC = middle upper arm circumference.

**Table 4:** Inter-anthropometrist reliability for repeated measurements of 89 children, stratified by quartile of measurement.

			Estimate (95% Confidence Interval)	
Quartile No.	Quartile Range	No. Subjects	TEM	%TEM, %
Height				
Quartile 1	60.5–77.8 cm	23	1.03 cm (0.73 to 1.33)	1.45 (1.03 to 1.87)
Quartile 2	77.9–90.6 cm	23	0.61 cm (0.43 to 0.78)	0.70 (0.50 to 0.90)
Quartile 3	90.7–97.2 cm	21	0.38 cm (0.27 to 0.50)	0.40 (0.28 to 0.53)
Quartile 4	97.3–116.4 cm	22	0.40 cm (0.28 to 0.52)	0.39 (0.28 to 0.51)
Weight				
Quartile 1	5.22–9.68 kg	23	0.11 kg (0.08 to 0.14)	1.34 (0.94 to 1.74)
Quartile 2	9.69–12.30 kg	22	0.10 kg (0.07 to 0.14)	0.91 (0.64 to 1.19)
Quartile 3	12.31–13.80 kg	22	0.07 kg (0.05 to 0.09)	0.55 (0.38 to 0.71)
Quartile 4	13.81–19.20 kg	22	0.09 kg (0.07 to 0.12)	0.59 (0.41 to 0.76)
MUAC				
Quartile 1	12.1–13.5 cm	24	0.22 cm (0.16 to 0.29)	1.73 (1.24 to 2.22)
Quartile 2	13.6–14.3 cm	21	0.19 cm (0.14 to 0.25)	1.40 (0.98 to 1.83)
Quartile 3	14.4–14.8 cm	22	0.22 cm (0.15 to 0.28)	1.49 (1.05 to 1.93)
Quartile 4	14.9–17.0 cm	22	0.23 cm (0.16 to 0.30)	1.48 (1.04 to 1.92)

TEM = technical error of measurement; %TEM = relative TEM.

**Table 5:** Inter-anthropometrist reliability of height measurements compared to length measurements.

	Estimate, % (95% Confidence Interval)	
Measurement	Height(N = 61)	Length(N = 28)
Mean	95.0 cm (92.9 to 97.0)	75.1 cm (71.6 to 78.5)
TEM	0.38 cm (0.31 to 0.45)	1.04 cm (0.76 to 1.32)
%TEM	0.40% (0.33 to 0.48)	1.37% (1.00 to 1.75)
Reliability (ICC)	0.998 (0.997 to 0.999)	0.987 (0.978 to 0.997)
Repeatability	1.06 cm (0.87 to 1.25)	2.87 cm (2.09 to 3.66)

We estimated the %TEM for the first of the 3 recorded measurements, as well as the median and mean of these 3 measurements (Table 6). We found that using the median of 3 measurements generally resulted in less error than taking either a single measurement or the mean measurement.

**Table 6:** Reliability of a single measurement, the median of 3 measurements, and the mean of 3 measurements.

	Relative Technical Error of Measurement, % (95% Confidence Interval)		
Measurement	Height	Weight	MUAC
INTRA-OBSERVER (N = 84)			
First of Three	0.43 (0.37 to 0.50)	0.68 (0.57 to 0.78)	1.46 (1.24 to 1.68)
Mean of Three	0.60 (0.51 to 0.69)	0.43 (0.37 to 0.50)	1.16 (0.99 to 1.34)
Median of Three	0.35 (0.30 to 0.41)	0.39 (0.33 to 0.45)	1.27 (1.08 to 1.46)
INTER-OBSERVER (N = 89)			
First of Three	0.96 (0.81 to 1.10)	0.96 (0.81 to 1.11)	1.50 (1.28 to 1.72)
Mean of Three	0.80 (0.68 to 0.92)	0.78 (0.66 to 0.91)	1.48 (1.26 to 1.69)
Median of Three	0.75 (0.64 to 0.86)	0.79 (0.67 to 0.91)	1.53 (1.30 to 1.76)

**Figure 3:**
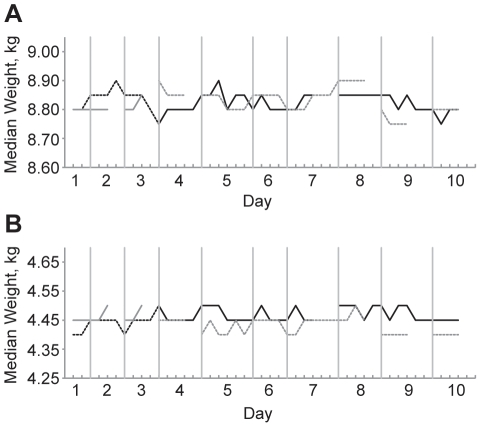
Reproducibility of scales in field conditions. Graphs show (A) the 9.0 kg test weight and (B) the 4.5 kg test weight, over the 10 days of the study. The 2 different scales are depicted in black or grey, and the 2 different test weight sets are depicted as dashed or solid lines. Test weights were measured after every 10^th^ child of the day, represented as a hash mark on the x-axis. Discontinuities in the lines indicate that the anthropometry team examined less children than the other team.

To determine the accuracy of the scales in field conditions, we weighed sets of test weights after every tenth child (Figure 3). We found that the maximum difference at any of the repeat measurements was only 0.15 kg, a number very similar to the intra- and inter-anthropometrist repeatability coefficients (Tables 2 and 3) and consistent with the manufacturer's insert. There appeared to be no change in the weight measurements over time in regression analyses adjusted for scale, test weight set, and anthropometry team: for each subsequent weighing, the measurement for the 4.5 kg test weight decreased by 0.0001 kg (95% CI −0.0006 to 0.0003) and the measurement for the 9.0 kg test weights decreased by 0.0002 kg (95% CI −0.0008 to 0.0004). We found no evidence for autocorrelation over time (Wooldridge test *p* = 0.44 for 4.5 kg test weight, and *p* = 0.52 for 9.0 kg test weight set).

**Figure 4:**
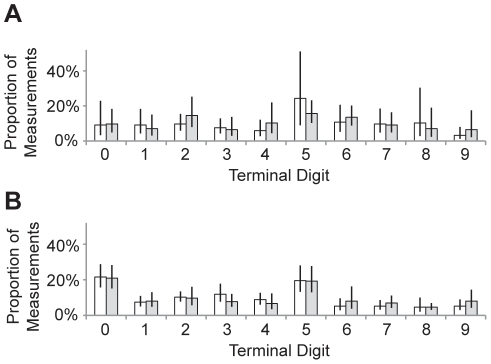
Terminal digit preference. The proportion of recorded measurements with each of the 10 possible terminal digits, shown for height (white) and MUAC (grey) measurements for (A) anthropometrist 1 and (B) anthropometrist 2. Error bars represent 95% confidence intervals, accounting for the clustered study design.

We tested for terminal digit preference in the 2 anthropometrists who had performed at least 100 measurements. We found evidence for terminal digit preference for the height measurements (*p*<0.0001 for each anthropometrist, χ^2^ test) and MUAC measurements (*p* = 0.48 and *p*<0.0001 for anthropometrists 1 and 2, respectively). Both anthropometrists frequently recorded 5 as the terminal digit, and the second anthropometrist also frequently recorded 0 (Figure 4).

## Discussion

We showed that rural community members without previous experience in anthropometry were able to take reliable anthropometric measurements after a short training exercise. Intra- and inter-anthropometrist reproducibility were relatively high for all metrics, though measurement error was slightly higher for smaller children than for larger children, and for length measurements compared to height measurements. The measurement error for weighing children was similar to that of weighing test weights.

Although growth monitoring of children would ideally be done by trained anthropometrists with formal health education, such individuals are usually not available in resource-poor settings. As an alternative, community members without formal training could be employed as anthropometrists [18], [19], [20]. However, the reliability of measurements made from community-drawn anthropometrists has not typically been reported in prior studies. We therefore attempted to address the reliability of community-drawn anthropometrists in a clinical trial setting in Ethiopia. As a first step, we assessed the agreement between anthropometrists and an independent observer in order to determine whether our anthropometrists would be able to accurately read the measurements from the anthropometry equipment. Anthropometry teams displayed very high agreement with the observers, suggesting that a brief training exercise was sufficient to teach our teams how to accurately use the equipment. We should point out, however, that the 6 anthropometrists in this study were selected from 22 potential candidates, many of whom were unable to adequately perform measurements after our training. Pre-testing of anthropometrists is therefore crucial when using community individuals with little training.

We also assessed intra- and inter-anthropometrist reproducibility, both of which were relatively high in this study. As expected, inter-anthropometrist measurement error was slightly greater than intra-anthropometrist error, and measurement error for height and weight were less than that for MUAC. The reliability estimates in this study were comparable to those found in previous studies in a variety of settings, suggesting that after appropriate training, community-drawn anthropometrists have the capacity to perform highly reliable measurements [14], [21].

Inter-anthropometrist error was greater for smaller children compared with larger children, and for length measurements compared with height measurements. This result is consistent with our experience in the field, where younger children were less cooperative and more difficult to measure. This result suggests that additional training could focus on techniques to accurately measure the youngest children, such as performing examinations quickly, and enlisting the help of guardians to comfort and stabilize the child, especially when measuring length. Even with this lack of precision for the youngest children, relative TEM was below 2% for the smallest quartile of all metrics, which is probably acceptable in most contexts.

In this study, taking the median of 3 serial height or weight measurements resulted in less measurement error than taking a single measurement, or taking the mean. However, the reduction in error was moderate: medians had approximately 10–20% lower measurement error than the single measurement. Therefore, although it appears reasonable to continue taking 3 measurements to reduce measurement error as much as possible, anthropometry teams could consider using a single measurement if taking multiple measurements per child became burdensome.

We repeatedly weighed test weight sets in order to rule out the possibility of bias in scale measurements over time. The measurements of the test weights did not change markedly over the course of the study. In fact, the minimum and maximum documented weights were only 0.15 kg apart, suggesting that the measurement error of the scale itself is about 0.15 kg in field conditions. That this degree of measurement error was similar to the intra-anthropometrist repeatability (0.15 kg) suggests that most of the intra-anthropometrist measurement error is due to the scale itself.

We found evidence for terminal digit preference among the anthropometrists, more so for height than for MUAC. This is a well-described phenomenon that can reduce precision of measurements [10], [22]. As has been found in other studies, the anthropometrists in this report seemed to prefer the numbers 0 and 5. The training program should address this concept in an attempt to improve measurement precision.

In conclusion, we found that rural community members were able to learn anthropometry techniques during a short training period. Height and weight measurements had high intra- and inter-anthropometrist reliability, and were more reproducible than measurements for MUAC. Measurement error was greater for smaller children than for larger children and for lengths compared to heights, likely because smaller children were less cooperative with the examination. This study suggests that height and weight measurements performed in the rural setting are appropriate outcomes for a clinical trial.
